# Environmental endocrine disruptors at the synapse: mechanisms linking chemical exposure to cognitive and behavioral dysfunction

**DOI:** 10.3389/fnbeh.2026.1821381

**Published:** 2026-05-22

**Authors:** Gergely Jocsak, David S. Kiss, Daiana Alymbaeva, Anna Diebald, Attila Zsarnovszky, Andrea Heinzlmann, Tibor Bartha

**Affiliations:** 1 Department of Physiology and Biochemistry, University of Veterinary Medicine, Budapest, Hungary; 2 Department of Physiology and Animal Health, Agrobiotechnology and Precision Breeding for Food Security National Laboratory, Institute of Physiology and Nutrition, Hungarian University of Agriculture and Life Sciences, Kaposvár, Hungary; 3 Department of Anatomy and Histology, University of Veterinary Medicine, Budapest, Hungary; 4 National Laboratory of Infectious Animal Diseases, Antimicrobial Resistance, Veterinary Public Health and Food Chain Safety, University of Veterinary Medicine, Budapest, Hungary

**Keywords:** long-term and short-term potentiation, memory, neural plasticity, neuroendocrine regulation, neuromodulation, neurotoxicity

## Abstract

Synaptic plasticity forms the basis for learning and memory processes, controlled by a finely tuned interaction of neurotransmitters, intracellular signaling pathways and hormonal modulators. Exogenous substances, especially endocrine disrupting chemicals (EDCs), can significantly disrupt these processes. EDCs such as bisphenols, phthalates, pesticides, PCBs/dioxins, heavy metals and flame retardants interfere with hormonal regulatory circuits, receptor systems and epigenetic programs. They alter glutamatergic transmission, disrupt calcium homeostasis, modulate signal transduction pathways and destabilize structural proteins. This results in functional disorders in long-term potentiation and depression, as well as morphological and functional changes in dendritic spines. In addition to functional deficits, EDCs lead to mitochondrial dysfunction, oxidative stress, inflammatory processes and impaired blood–brain barrier (BBB) integrity. These mechanisms manifest themselves in cognitive impairments, behavioral abnormalities and increased susceptibility to neuropsychiatric disorders. Exposure during sensitive developmental phases is particularly critical, as it can be passed on transgenerationally *via* epigenetic changes. Integrative analysis shows that heterogenous EDCs undermine neural plasticity *via* convergent molecular mechanisms. They, therefore, pose a significant challenge to public health and prevention. A better understanding of these processes is essential to strengthen regulatory measures and minimize long-term risks to cognitive function and behavior.

## 1 Introduction

Synaptic plasticity (SP) describes the ability of the central nervous system (CNS) to dynamically adapt to functional demands by modifying synaptic strength, connectivity, and, in some contexts, the number of synaptic contacts in an activity- and experience-dependent manner (Citri and Malenka, 2008). It constitutes the cellular and molecular basis of learning and memory and encompasses both functional forms of plasticity, such as long-term potentiation (LTP) and long-term depression (LTD), and structural remodeling of neuronal connectivity (Marrone and Petit, 2002; Zhang et al., 2003). These processes are tightly regulated by neurotransmitters, intracellular signaling pathways, and endocrine modulators, particularly sex steroids and thyroid hormones.

Because synaptic plasticity is deeply embedded in endocrine regulation, it is inherently vulnerable to endocrine-disrupting chemicals (EDCs), a diverse class of natural and synthetic compounds that interfere with hormonal signaling pathways and thereby alter development, metabolism, reproduction, and neural function (Gore, 2010; Somogyi et al., 2011). EDCs are widespread in the environment, may accumulate in organisms, and often exert biological effects even at low, environmentally relevant doses (Weiss, 2011). Exposure during sensitive developmental periods, including pregnancy and childhood, is of particular concern because these windows coincide with critical stages of neural circuit formation and synaptic maturation.

A broad spectrum of EDCs including bisphenols, phthalates, pesticides, polychlorinated biphenyls (PCBs), dioxins, heavy metals, flame retardants (FRs), and polycyclic aromatic hydrocarbons (PAHs) has been shown to impair neural plasticity, cognitive function, and behavior. Despite their chemical diversity, increasing evidence suggests that these substances converge on a limited number of shared biological mechanisms, including receptor-mediated signaling disturbances, altered excitatory and inhibitory neurotransmission, oxidative stress, mitochondrial dysfunction, and epigenetic reprogramming. Although several reviews have examined EDC effects on hypothalamic function and neuroendocrine regulation (Maia et al., 2025; Parent et al., 2025), comparatively few have focused on the structure and cellular function of extrahypothalamic regions of the CNS. The central question of the present review is therefore which common molecular mechanisms different EDC classes use to disrupt synaptic plasticity outside the hypothalamus, and how these disturbances translate into cognitive and behavioral dysfunction.

In this review, we propose a unifying mechanistic framework in which heterogeneous EDCs disrupt synaptic plasticity through five core axes:

1. Receptor-mediated disruption.
2. Calcium signaling and synaptic machinery interference.
3. Oxidative stress and mitochondrial dysfunction.
4. Epigenetic reprogramming.
5. Network-level and barrier dysfunction.

**Figure 1:**
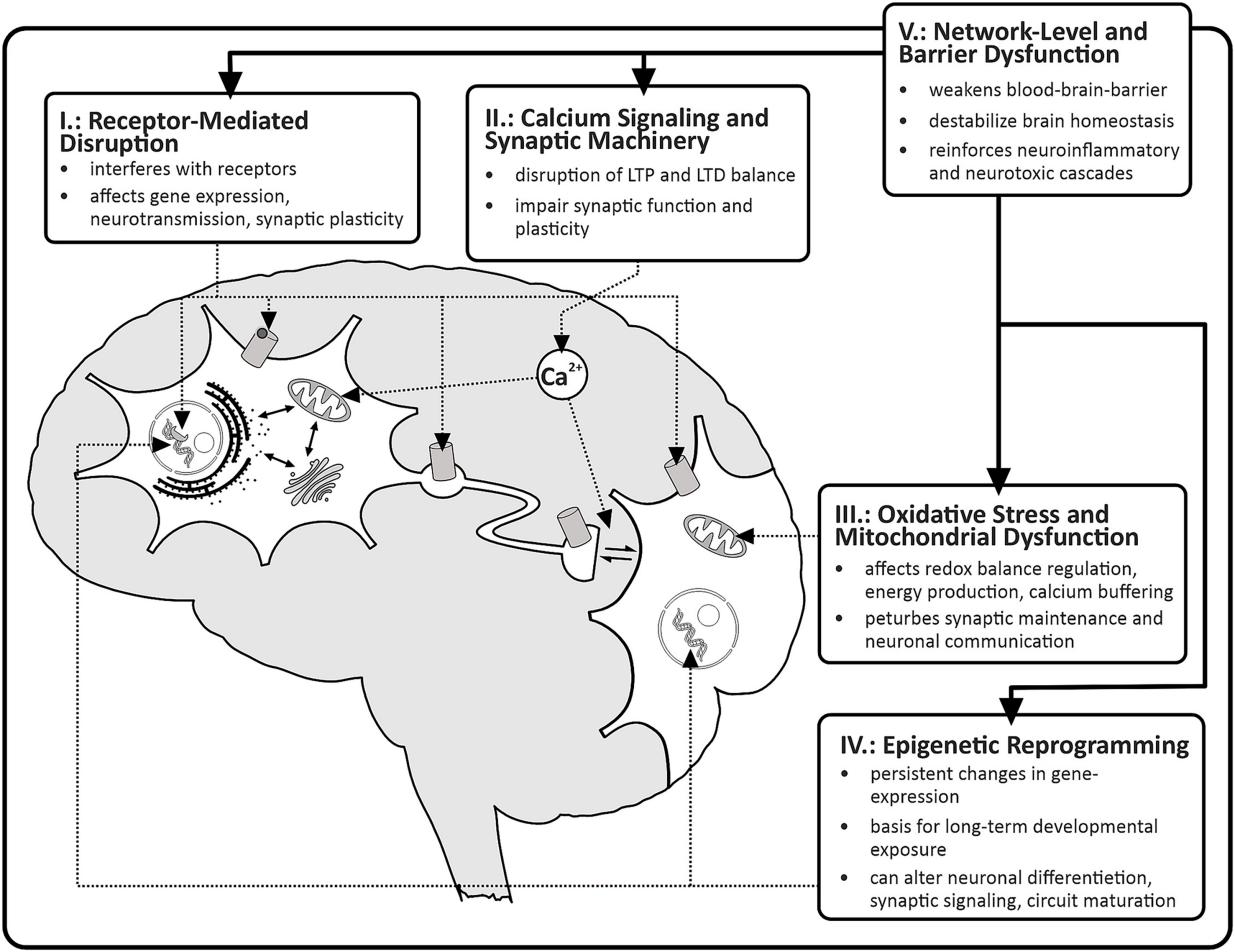
Mechanistic axes of endocrine disruption in neurons. Endocrine-disrupting chemicals (EDCs) impair neuronal function through multiple, interconnected mechanistic pathways. Axis I (receptor-mediated disruption) involves interference with nuclear and membrane-associated receptors, leading to altered intracellular signaling and dysregulated gene expression. Axis II (calcium signaling and synaptic machinery) reflects disturbances in Ca^2+^ homeostasis, resulting in impaired kinase/phosphatase activity, disrupted receptor trafficking, and imbalance between long-term potentiation (LTP) and long-term depression (LTD). Axis III (oxidative stress and mitochondrial dysfunction) represents a convergent downstream mechanism characterized by increased reactive oxygen species (ROS) production, reduced ATP generation, and impaired calcium buffering, ultimately compromising neuronal survival and synaptic maintenance. Axis IV (epigenetic reprogramming) encompasses persistent alterations in DNA methylation, histone modification, and non-coding RNA expression, leading to long-term changes in neuronal differentiation, synaptic signaling, and circuit maturation. Axis V (network-level and barrier dysfunction) highlights disruption of the neurovascular unit and blood–brain barrier integrity, facilitating the entry of peripheral mediators and promoting neuroinflammation and circuit-level dysfunction. Together, these mechanisms converge to produce synaptic impairment, neuronal dysfunction, and long-term alterations in brain function and behavior.

This framework shifts the perspective from a substance-centered to a mechanism-centered understanding, highlighting convergent biological targets that link endocrine disruption to altered synaptic integrity, impaired plasticity, and ultimately cognitive and behavioral abnormalities (see Figure 1).

## 2 Methods

This review was conducted as a focused narrative synthesis aimed at identifying shared mechanisms by which EDCs impair synaptic plasticity in extrahypothalamic regions of the central nervous system. The review was structured around five predefined mechanistic axes: receptor-mediated disruption, calcium signaling and synaptic machinery interference, oxidative stress and mitochondrial dysfunction, epigenetic reprogramming, and network-level and barrier dysfunction.

Relevant literature was identified through targeted searches of PubMed/MEDLINE, Web of Science, and Scopus, supplemented by manual screening of reference lists from key reviews and primary studies. Search terms combined concepts related to synaptic plasticity, cognition, and behavior with terms related to endocrine disruption and major EDC classes. Core search words included combinations of *“synaptic plasticity,” “long-term potentiation,” “long-term depression,” “dendritic spine,” “hippocampus,” “cortex,” “amygdala,” “cognition,” and “behavior” with “endocrine disrupt,” “bisphenol,” “phthalate,” “pesticide,” “PCB,” “dioxin,” “heavy metal,” “lead,” “cadmium,” “arsenic,” “flame retardant,” “PBDE,”* “*organophosphate flame retardant*,” *“PAH,” “benzo[a]pyrene,” “zearalenone,” “receptor,” “calcium signaling,” “oxidative stress,” “mitochondria,” “epigenetic,” “microglia,” “astrocyte,”* and *“blood–brain barrier.”*

Studies were considered eligible if they addressed EDC effects on synaptic plasticity, neurotransmission, dendritic spine remodeling, calcium signaling, oxidative stress, mitochondrial function, epigenetic regulation, neuroinflammation, blood–brain barrier integrity, or behavioral and cognitive outcomes relevant to CNS function. Human epidemiological studies, animal experiments, *in vitro* mechanistic studies, and selected review articles were included when they contributed directly to the mechanistic framework of the manuscript. Priority was given to studies examining brain regions outside the hypothalamus, particularly the hippocampus, cortex, amygdala, and related cognitive and affective circuits. Studies focused exclusively on peripheral endocrine effects without neural endpoints were not prioritized. Because of the marked heterogeneity of study designs, exposure paradigms, species, endpoints, and analytical methods, the evidence was synthesized qualitatively rather than by formal meta-analysis.

No lower publication-year limit was applied; database searches were updated through March 2026.

## 3 Theoretical basis of synaptic plasticity and endocrine vulnerability

Neuroplasticity is a fundamental property of the CNS that enables neural circuits to adapt to activity and experience by modifying synaptic strength, connectivity, and, in some contexts, structural organization (Nugent et al., 2023; Weiss, 2007; Zsarnovszky et al., 2024). This capacity persists throughout life and includes both the remodeling of synaptic contacts and, in selected brain regions such as the hippocampus, continued neurogenesis. Synaptic plasticity is commonly divided into two closely interconnected forms: functional synaptic plasticity, which refers to long-lasting changes in synaptic efficacy, and structural or morphological plasticity, which involves dynamic changes in dendritic spine density and morphology. These processes constitute a central cellular basis of learning and memory in both humans and experimental animals (Batool et al., 2019; Bettio et al., 2020; Hyun and Ka, 2024; Jin and Karlstedt, 2025; Ogiue-Ikeda et al., 2008; Seralini and Jungers, 2021; Sha et al., 2023; Weiss, 2011).

At the molecular level, functional synaptic plasticity is critically regulated by glutamatergic signaling, particularly through N-methyl-D-aspartate (NMDA) receptors. Activation of these receptors triggers postsynaptic Ca^2+^ influx, and the magnitude and duration of this signal determine the direction of plasticity (Jin and Karlstedt, 2025; Rodrigues et al., 2024). A rapid and pronounced rise in intracellular Ca^2+^ favors LTP by activating kinases such as Ca^2+^/calmodulin-dependent protein kinase II and promoting insertion of *α*-Amino-3-hydroxy-5-methyl-4-isoxazolepropionic acid, better known as (AMPA) receptors into the postsynaptic membrane. In contrast, a more moderate but sustained Ca^2+^ increase promotes LTD through activation of phosphatases such as calcineurin and protein phosphatase 1, resulting in AMPA receptor internalization. Structural plasticity is tightly coupled to these functional changes, as LTP is generally associated with spine enlargement or increased spine density, whereas LTD is linked to spine shrinkage and synaptic weakening (Jin and Karlstedt, 2025; Rodrigues et al., 2024).

These plastic processes are embedded in endocrine regulation. Sex steroids, thyroid hormones, and glucocorticoids modulate synaptic transmission, dendritic spine dynamics, neuronal excitability, and circuit maturation, particularly in plasticity-relevant regions such as the hippocampus and hypothalamus (Damiano et al., 2025; Dos-Santos et al., 2023; Dsouki et al., 2024; Ogiue-Ikeda et al., 2008; Pang and Han, 2012; Parent et al., 2011; Weiss, 2007). This close integration of synaptic and endocrine signaling renders the CNS particularly vulnerable to EDCs, which are exogenous substances or mixtures that interfere with endocrine function and thereby cause adverse effects in exposed organisms or their offspring (Damiano et al., 2025; Gilbert et al., 2020; Klyubin et al., 2022; Schug et al., 2015). Because exposure frequently occurs during highly sensitive developmental windows, including fetal life and early childhood, EDC-induced disturbances of receptor signaling, calcium homeostasis, oxidative balance, and gene regulation can have persistent consequences for neural plasticity, cognition, and behavior (Parent et al., 2011; Schug et al., 2015; Walker and Gore, 2017; Weiss, 2007).

## 4 Overview of major endocrine disruptor classes

### 4.1 Bisphenols

Bisphenols are among the most intensively studied EDCs because their widespread industrial use means they are virtually ubiquitous in the environment and in everyday human life. The most prominent member of this group of substances is bisphenol A (BPA), a synthetic xenoestrogen that has been used in plastics and resin production for decades (Weiss, 2007). Products such as baby bottles, the inner coatings of food cans, microwave dishes and even dental sealants are important sources of human exposure (Weiss, 2011, 2007). Although regulatory measures in recent years have led to a partial decline in BPA use, structurally related substitutes such as bisphenol S (BPS), bisphenol F (BPF) and bisphenol AF (BPAF) have been increasingly introduced (Dias et al., 2025). These substances were originally intended to serve as “safe alternatives,” but there is now growing evidence that they too have neurotoxic and endocrine-disrupting properties. In experimental studies, exposure to BPA and BPS in zebrafish led to premature hypothalamic neurogenesis even at low doses (Kinch et al., 2015; Walker and Gore, 2017). This makes it clear that the substitution principle does not necessarily lead to a reduction in health risks in this case. A well-known example of an estrogenic EDC is diethylstilbestrol (DES), which was prescribed to pregnant women in the mid-20th century to prevent miscarriages (Parent et al., 2011; Soyer-Gobillard et al., 2025).

The consequences of the widespread use of bisphenols are serious: even in the first generation, not only metabolic disorders such as obesity and altered hippocampal development were observed, but also severe neuropsychiatric disorders were observed in the offspring. Children who were exposed in utero developed schizophrenia (22.9%), depression and bipolar disorder (34.4%), anxiety disorders and suicide attempts at a high prevalence (Soyer-Gobillard et al., 2025). This example clearly illustrates how profoundly EDCs interfere with the development of the nervous system and how severe the long-term consequences can be.

The significance of bisphenols for neuroplasticity is therefore by no means a theoretical question, but a central aspect of public health. The following section explains the key mechanisms by which bisphenols modulate neural plasticity, thereby causing both structural and functional changes in the brain.

### 4.2 Phthalates

Phthalates are a large group of synthetic chemicals derived from petrochemical by-products and are mainly used as plasticizers in industry to modify the physical properties of plastics, especially polyvinyl chloride (PVC) (Wormuth et al., 2006). Due to this property, they are widely used in numerous consumer and industrial products. They are found in cosmetics such as perfumes, hair sprays, soaps and nail varnishes, in medical devices such as catheters and tubes, in food contact materials such as films and packaging, and even in pesticides, where they are added as “inactive” components. This ubiquitous presence means that the general population is continuously exposed, with the main routes of exposure being through food, inhalation and skin contact. Because several widely used phthalates are capable of disrupting endocrine signaling pathways, they have been officially recognized as endocrine disruptors (Demeneix and Slama, 2019).

Among the best-studied representatives of this class of substances are dibutyl phthalate (DBP), di(2-ethylhexyl) phthalate (DEHP) and benzyl butyl phthalate (BBP). Their biologically active metabolites are produced by hydrolysis processes, such as mono-n-butyl phthalate (MnBP) from DBP or mono-2-ethylhexyl phthalate (MEHP) from DEHP. Diisononyl phthalate (DINP) is also highly relevant (Koch et al., 2004; Saravanabhavan and Murray, 2012; Silva et al., 2007). Phthalates were originally often described as xenoestrogens, but research has shown that their main endocrine effects are largely antiandrogenic. Animal studies have shown that phthalates inhibit testosterone production in the testicles (Howdeshell et al., 2008; Swan et al., 2005). Perinatal exposure can impair sexual differentiation in humans, as evidenced by, among other things, a shortened anogenital distance in male newborns (Swan et al., 2005). In addition to effects on the androgen system, data show that phthalate metabolites were associated with altered thyroid function in preschool children (Morgenstern et al., 2017).

In sum, the neurotoxic effects of phthalates are based on complex molecular and cellular processes involving nuclear receptors, signal transduction pathways, stress responses and synaptic plasticity.

### 4.3 Pesticides

Pesticides, including organophosphates, organochlorines, and organotin compounds, are important environmental toxicants, and several members of these classes also meet endocrine-disruptor criteria because they interfere with hormonal signaling pathways. Synaptic function and plasticity appear to be particularly vulnerable targets of pesticide exposure. For example, chlorpyrifos exposure dysregulates synaptic plasticity in rat hippocampal neurons, reducing dendritic spine density and decreasing expression of synaptophysin, PSD-95, GluN1, and GluA1. Organotin compounds can also directly impair synaptic communication: in mouse cerebral cortex, tri-, di-, and monobutyltin altered cholinergic synaptic parameters relevant to neurotransmission. Together, these findings indicate that pesticides are not only general neurotoxicants, but can also interfere directly with the molecular and synaptic processes that support neural plasticity (Bergman et al., 2012; Zhou et al., 2023).

### 4.4 PCBs and dioxins

PCBs and dioxins are a group of persistent organic pollutants (POPs) that are causing concern worldwide. PCBs are a class of industrial chemicals consisting of up to 209 different congeners (Parent et al., 2011; Schug et al., 2015). They were used in a wide range of applications from the 1930s to the 1970s, for example as cooling lubricants, in hydraulic fluids, as plasticizers or FRs. Although production was discontinued in many countries – for example, in the United States as early as 1979 – PCBs continue to be ubiquitous in the environment due to their high stability, lipophilicity and longevity. They accumulate in the fatty tissue of animals and humans and bioaccumulate in the food chain, leading to continuous exposure (Parent et al., 2011; Schug et al., 2015; Walker and Gore, 2017).

Dioxins, which are chemically described as polychlorinated dibenzodioxins and dibenzofurans, are not deliberately produced substances, but are unintentional by-products of combustion processes or the manufacture of certain chlorinated chemicals. Of particular relevance are tetrachlorodibenzo-p-dioxin (TCDD) and tetrachlorodibenzofuran, which are of great toxicological significance due to their structural similarity to dioxin-like PCBs and their ability to bind to the same cellular receptor (Poland et al., 1976; Yavuz et al., 2023). The neurotoxicity of PCBs and dioxins is particularly critical during brain development (Weiss, 2007). The development of the central nervous system is highly dependent on intact regulation by thyroid and sex hormones. Disruptions in these systems can have far-reaching consequences for synaptic connectivity patterns. PCBs and dioxins interfere with hormonal signaling pathways and thus also impair synaptic plasticity (Parent et al., 2011). Since synaptic plasticity forms the basis for learning processes, interference by EDCs in these mechanisms leads to permanent changes in neural networks. In numerous studies, PCBs have therefore been cited as the most convincing evidence for the link between exposure to endocrine disruptors and impaired cognitive function (Schug et al., 2015).

### 4.5 Heavy metals

Heavy metals are a significant group of environmental contaminants that are closely linked to neurotoxic and endocrine disorders. Among the most studied representatives are arsenic (As), lead (Pb) and cadmium (Cd), which enter the environment through mining, metal smelting, industrial emissions and other anthropogenic processes (Schug et al., 2015; Zsarnovszky et al., 2024). Unlike many organic endocrine disruptors, heavy metals are characterized by their ability to accumulate in almost all tissues, where they persist for long periods of time and influence biochemical functions in a variety of ways. Arsenic is classified as the most dangerous toxic substance by the Agency for Toxic Substances and Disease Registry (ATSDR), underscoring its high relevance to environmental and health issues (Zsarnovszky et al., 2024). In addition to classic heavy metals, organotin compounds such as dibutyl tin or tributyl tin are also discussed as problematic substances, which are mainly used in plastics as stabilizers or as fungicides in agriculture. Their persistence and mechanism of action in the central nervous system also place them in the category of endocrine disruptors.

### 4.6 Other substances

In addition to the main groups of endocrine disruptors discussed in detail above, there are other substances that also have neurotoxic potential and can interfere with synaptic plasticity. Their effects on synaptic plasticity have been scientifically proven, which is why these substances will also be presented in condensed form in this paper.

Mycotoxins form a second group of endogenous environmental toxins whose effects on synapses are increasingly being investigated. These secondary metabolites produced by molds are of global relevance, particularly due to their presence in contaminated food and feed. Among them, zearalenone (ZEN) plays a special role, as it can directly interfere with hormonal signaling pathways as a phytoestrogen due to its structural similarity to 17-β-estradiol (Jocsak et al., 2019; Kiss et al., 2018).

FRs are a very heterogeneous group of synthetic chemicals used in a wide range of industrial and household products to reduce flammability (Rojas et al., 2025). These substances are particularly relevant because they accumulate in the environment and are detectable in organisms. Among the most prominent representatives are polybrominated diphenyl ethers (PBDEs), which are characterized by their persistence and lipophilic nature. Due to regulatory restrictions, PBDEs are increasingly being replaced by organophosphate flame retardants (OPFRs), which are by no means harmless (Damiano et al., 2025; Dsouki et al., 2024; Rojas et al., 2025). Both groups exhibit endocrine disrupting properties and also have pronounced neurotoxic effects.

Pharmaceuticals are another heterogeneous group that has become relevant in ecological contexts, as they can enter aquatic systems *via* wastewater and are detectable there in trace concentrations (Seralini and Jungers, 2021). Synthetic sex hormones such as DES are particularly relevant, but so are anticonvulsants such as valproic acid (VPA) and antidepressants such as citalopram. Natural steroids such as testosterone and their analogues can also have neurotoxic effects in pathologically elevated concentrations (Chu et al., 2015; Pang and Han, 2012; Sass and Wörtwein, 2012; Soyer-Gobillard et al., 2025).

Finally, PAHs are a group of ubiquitous pollutants that are mainly produced by the incomplete combustion of fossil fuels and are therefore widespread in urban environments. They are found in car exhaust fumes, coal tar and numerous other sources and are also classified as endocrine disruptors due to their persistence and biological effects (Das et al., 2019; Schug et al., 2015; Tang et al., 2011). Benzo[a]pyrene (BaP) and anthracene have been studied particularly intensively.

## 5 Mechanistic axis I: receptor-mediated disruption

A primary mode of EDC action involves interference with nuclear and membrane-associated receptors that regulate gene expression, neurotransmission, and synaptic plasticity. Through these receptor-mediated mechanisms, EDCs can rapidly alter intracellular signaling while also reprogramming transcriptional pathways required for neuronal differentiation, synaptic maintenance, and cell survival.

Bisphenols are prototypical examples of this mode of action. BPA acts as a xenoestrogen by binding estrogen receptors (ER), including ERα and ERβ, and by activating both genomic and rapid non-genomic signaling pathways. In hippocampal glutamatergic neurons, BPA binds membrane-associated ERα and, even at low concentrations (10–100 nM), rapidly increases intracellular Ca^2+^ and activates ERK/MAP kinase signaling (Gould et al., 1998; Ogiue-Ikeda et al., 2008). Because estrogen receptor signaling is tightly integrated with glutamatergic plasticity, such dysregulation has important downstream consequences for synaptic function. Bisphenols also interfere with glutamate receptor-dependent processes, and disturbances in AMPA and NMDA receptor expression, phosphorylation, or membrane localization are particularly damaging because these receptors are essential for excitatory synaptic transmission and plasticity (Hyun et al., 2022; Wang et al., 2021). ZEN likewise competes with endogenous ligands for estrogen receptor binding and thereby disrupts hormone-sensitive developmental and neuronal signaling pathways (Frizzell et al., 2011; Takemura et al., 2007).

Phthalates predominantly act through nuclear receptor systems. DBP and related compounds are best known for their antiandrogenic activity, but they also interact with peroxisome proliferator-activated receptors, particularly peroxisome proliferator-activated receptor gamma (PPARγ), and with the aryl hydrocarbon receptor (AhR), thereby affecting transcriptional regulation, apoptosis, and neurodevelopmental programming (Kajta et al., 2009; Wójtowicz et al., 2017). Although phthalates are often less directly linked than bisphenols to fast synaptic signaling, their capacity to alter receptor-dependent transcriptional programs makes them important modulators of neuronal maturation and plasticity.

PCBs and dioxins exert especially broad receptor-mediated effects. A central mechanism is activation of the AhR. Coplanar PCB congeners such as CB-77 and dioxins such as TCDD bind AhR with high affinity, leading to dysregulated gene expression and increased neuronal apoptosis (Gilbert and Crofton, 1999; Parent et al., 2011). In parallel, these compounds disrupt thyroid hormone signaling, a pathway essential for brain development and synaptic maturation. PCBs reduce circulating thyroxine, interfere with hepatic and cerebral thyroid hormone metabolism, compete with thyroxine (T4) for binding to transthyretin, and can interact directly with thyroid hormone receptors, acting in a context-dependent agonistic or antagonistic manner [a8] (Dsouki et al., 2024; Schug et al., 2015; Zoeller et al., 2000). Developmental exposure to Aroclor 1254 (a PCB mixture) alters expression of thyroid hormone-responsive genes such as RC3/neurogranin and myelin basic protein, produces morphological changes consistent with hypothyroid-like neurodevelopment, and impairs dendritic growth and mossy fiber organization in the hippocampus. PCBs and dioxins also affect neurotransmitter systems more directly: dioxin-like compounds reduce acetylcholinesterase expression, developmental PCB exposure alters dopamine levels (essential for the regulation of motivation, reward and motor processes) and turnover, and Aroclor 1,254 decreases somatodendritic vasopressin release (Gilbert and Crofton, 1999; Parent et al., 2011). These receptor- and hormone-dependent disturbances are functionally reflected in electrophysiological deficits, including reduced hippocampal and dentate gyrus LTP after developmental exposure to Aroclor 1254 or CB-77 (Gilbert and Crofton, 1999; Ogiue-Ikeda et al., 2008; Zoeller et al., 2000). PAHs converge on related mechanisms through AhR activation; BaP, for example, reduces glutamate concentration and glutamate receptor expression, thereby weakening hippocampal LTP and impairing learning and memory, while anthracene further disrupts cholinergic signaling through acetylcholinesterase inhibition which leads to the prolonged presence of acetylcholine in the synaptic cleft (Brown et al., 2007; Gilbert and Crofton, 1999; Jacobson and Jacobson, 1996; Lyu et al., 2020; Olasehinde and Olaniran, 2022; Zoeller et al., 2000).

Heavy metals and several pesticide classes also disrupt receptor-mediated neurotransmission. Arsenic impairs glutamatergic signaling by reducing expression of the NR2A subunit of the NMDA receptor in the hippocampus, a change associated with impaired LTP and deficits in learning and memory (Luo et al., 2012, 2009; Zsarnovszky et al., 2024). It also increases extracellular glutamate and disrupts cystine/glutamate transport, thereby promoting excitotoxicity and neuronal injury (Nelson-Mora et al., 2018; Singh et al., 2016). More broadly, pesticides interfere with cholinergic, glutamatergic, and GABAergic systems that maintain excitatory/inhibitory balance. In mice, tributyltin metabolites have been shown to interfere with cholinergic signaling pathways in the cerebral cortex, resulting in disturbed synaptic transmission (Kobayashi et al., 1996). Dieldrin reduces both GABA-A and NMDA receptor function in cerebellar granule cells, while CPO increases the ratio of GABA-A receptor-mediated to AMPA receptor-mediated currents in CA1 pyramidal neurons, shifting network activity toward inhibition (Babot et al., 2007; López-Merino et al., 2022). This disturbance of excitatory/inhibitory balance is increasingly recognized as a core mechanistic feature linking EDC exposure to neurodevelopmental dysfunction. Chronic pesticide exposure also persistently over activates the MAPK/ERK pathway in the hippocampus. Organochlorines such as chlordane, dieldrin, and endosulfan, as well as organophosphates such as chlorpyrifos and chlorpyrifos oxon, induce sustained ERK activation and disrupt mGluR-dependent LTD; for chlordane, pharmacological inhibition of ERK signaling rescues this plasticity deficit, supporting a causal relationship between receptor-linked ERK dysregulation and synaptic dysfunction (López-Merino et al., 2022). Organotin compounds add a further layer of disruption by altering inhibitory synaptic currents and cholinergic transmission, suggesting broader neuromodulatory instability (Kobayashi et al., 1996; Yavuz et al., 2023).

FRs and related compounds target multiple receptor systems relevant to neuronal development, synaptic stability, and circuit maturation. Certain OPFRs, including tris(2-chloroethyl) phosphate (TCEP) and triphenyl phosphate (TPP), increase extracellular glutamate and thereby promote excitotoxic stress. At the nuclear receptor level, tetrabromobisphenol A (TBBPA) suppresses PPARγ expression in cortical neurons, whereas bis-(2-ethylhexyl)-phenyl phosphate (BEHPP) acts as an antagonist of the retinoic acid receptor RARα, two mechanisms that can profoundly interfere with neuronal differentiation and synaptic organization (Jia et al., 2022; Wojtowicz et al., 2014). Recent primary studies further show that FRs also disrupt receptor-defined neuromodulatory systems that guide synapse formation and socioemotional circuit development. Maternal exposure to a mixture of OPFRs altered development of the mesencephalic dopamine system in fetal rats, producing morphological changes in the putative ventral tegmental area and substantia nigra, reducing dopaminergic cell-cluster volume in males, and disrupting dopaminergic axogenesis (Newell et al., 2023b). More recent work also indicates sex- and chemical-specific disruption of social-brain targets involving oxytocin receptor- and serotonin-related signaling after perinatal FireMaster 550 exposure (Krentzel et al., 2021; Schkoda et al., 2024). In parallel, several FRs, including BDE-209, TBBPA, TPP, and hexabromocyclododecane (HBCD), reduce expression of synaptic proteins such as synaptophysin and PSD-95 in the hippocampus, indicating that receptor-mediated developmental disruption is translated into structural instability at the synapse. Reduced expression of these proteins is closely associated with impaired learning and memory as well as with psychiatric vulnerability (Al-Mousa and Michelangeli, 2014; Li et al., 2017). Together, these findings strengthen the view that FRs disrupt monoaminergic, neuropeptidergic, and synaptic regulatory pathways that are essential for synaptic organization, stability, and behavioral function (Kim et al., 2025; Vuong et al., 2020).

Selective serotonin reuptake inhibitors (SSRIs) such as norfluoxetine or citalopram modulate serotonergic neurotransmission, thereby altering spatial learning and memory performance (Sass and Wörtwein, 2012). Norfluoxetine also acts as a stimulatory agent for steroidogenesis in the brain, which influences GABA(A) receptor-mediated transmission and can result in profound changes in the neuronal excitation balance (Pinna et al., 2009, 2006). Synthetic glucocorticoids such as dexamethasone, in turn, affect the phosphorylation status of AMPA receptors in the human limbic system. Since these receptors significantly determine the strength of synaptic transmission, the effects on synaptic plasticity are considerable (Lopes et al., 2016). In combination, pharmaceuticals show that even therapeutically effective substances can contribute to the disruption of synaptic processes in unintended contexts.

Beyond receptor- and signaling-related abnormalities, EDCs also induce structural remodeling of synapses, particularly at the level of dendritic spines, the principal postsynaptic sites of excitatory neurotransmission. Bisphenol exposure illustrates the context-dependent nature of these effects. *In vitro*, BPA (10 nM) and DES (1 nM) increase spine density in the CA1 hippocampus of rats, particularly by increasing the proportion of thin, unstable spines, suggesting enhanced but less stable synaptic connectivity. *In vivo*, however, moderate BPA exposure reduced spine density and prevented estradiol-induced synaptogenesis in the hippocampus and prefrontal cortex, indicating that xenoestrogenic effects on synaptic structure are dose-, context-, and region-dependent (Ogiue-Ikeda et al., 2008; Rodrigues et al., 2024; Weiss, 2007). Similar structural vulnerability is observed with other EDCs, including FRs such as BDE-209, TBBPA, and TPP, which downregulate synaptic proteins such as synaptophysin and PSD-95 in the hippocampus, thereby weakening synaptic architecture and potentially limiting synapse stabilization through reduced brain-derived neurotrophic factor (BDNF) support (Li et al., 2017; Zhong et al., 2021).

Taken together, these findings show that receptor-mediated disruption is a unifying upstream mechanism across chemically diverse EDC classes. By targeting estrogen, androgen, AhR, thyroid hormone, PPAR, retinoic acid, cholinergic, glutamatergic, and GABAergic signaling systems, EDCs alter the transcriptional and electrophysiological programs that sustain synaptic integrity, plasticity, and neuronal survival. These receptor-level disturbances then propagate into the downstream abnormalities in calcium homeostasis, oxidative stress, mitochondrial function, and network stability described in the following mechanistic axes.

## 6 Mechanistic axis II: calcium signaling and synaptic machinery

Calcium signaling is a central determinant of the directionality of synaptic plasticity, and EDC-induced disturbances in calcium homeostasis can therefore directly disrupt the balance between LTP and LTD. Because calcium dynamics regulate kinase- and phosphatase-dependent pathways that control receptor trafficking and synaptic strength, perturbations in intracellular Ca^2+^ signaling can profoundly impair synaptic function and plasticity.

Several classes of EDCs interfere with calcium signaling through distinct yet convergent mechanisms. Bisphenols, for example, induce rapid increases in intracellular Ca^2+^ *via* membrane-associated estrogen receptor signaling, thereby mimicking endogenous estrogenic effects, but in a dysregulated manner that may disturb normal synaptic modulation (Ogiue-Ikeda et al., 2008).

Heavy metals also target calcium-dependent signaling pathways. Cadmium reduces the availability of calmodulin, a calcium-binding protein essential for the activity of calcium/calmodulin-dependent kinases, which play a crucial role in neurotransmitter release and thus in the regulation of synaptic efficiency (Schug et al., 2015). Lead poses a particular risk because it can act as a calcium mimetic, thereby interfering with Ca^2+^-dependent signaling pathways, compromising the fidelity of synaptic transmission, and severely disrupting early neurodevelopment (Florea et al., 2013; Sharma et al., 2015).

FRs likewise exert pronounced effects on intracellular calcium regulation and calcium-dependent synaptic machinery. TBBPA, for instance, induces dose-dependent cytotoxicity in cerebellar granule cells, an effect attributed to disturbed calcium currents together with increased oxidative stress (Al-Mousa and Michelangeli, 2014; Reistad et al., 2007; Zieminska et al., 2017). PBDEs also impair calcium homeostasis and promote mitochondrial dysfunction and apoptotic processes, while HBCD inhibits the sarco/endoplasmic reticulum Ca^2+^-ATPase (SERCA) pump, a key regulator of intracellular calcium storage and signaling. More broadly, FR-mediated impairment of calcium pumps such as SERCA further destabilizes intracellular calcium dynamics and contributes to synaptic dysfunction. Additional primary evidence links these molecular disturbances to electrophysiological plasticity and ion-channel regulation. In neonatal mice, TBBPA caused a small reduction in post-tetanic potentiation, although overall LTP effects remained modest, indicating that even brief developmental exposure can perturb early forms of activity-dependent synaptic strengthening (Dingemans et al., 2007; Hendriks et al., 2015). Complementing this physiological evidence, developmental organophosphate ester exposure produced sex-specific hippocampal transcriptomic changes enriched for cation-transport pathways, including voltage-gated potassium and calcium channel genes and subunits, supporting the conclusion that FRs also disturb intrinsic excitability and calcium-dependent synaptic machinery at the gene-regulatory level (Newell et al., 2023b). Together, these findings indicate that FRs disrupt calcium homeostasis across multiple levels, from intracellular storage and calcium currents to ion-channel regulation and activity-dependent synaptic plasticity.

PCBs represent another important class of EDCs that disrupt calcium homeostasis at multiple levels. Non-coplanar PCB congeners cause a slow but sustained rise in intracellular calcium concentrations in cerebellar granule cells and cortical neurons, primarily through inhibition of mitochondrial and microsomal calcium uptake [a8]. Even at concentrations that do not produce overt cytotoxicity, PCBs can induce the translocation of protein kinase C (PKC) to the membrane, a process associated with PKC activation and subsequent disruption of downstream signaling pathways. In addition, PCBs activate ryanodine receptors, thereby further amplifying intracellular calcium release and triggering caspase-dependent cell death in embryonic hippocampal neurons (Schug et al., 2015; Yavuz et al., 2023). More generally, PCB-induced impairment of mitochondrial and endoplasmic reticulum calcium handling, together with ryanodine receptor activation, can lead to sustained intracellular calcium elevation, excitotoxic stress, and neuronal injury (Howard et al., 2003; Wong et al., 1997).

Taken together, these chemically diverse insults converge on a common pathogenic mechanism: the destabilization of calcium-dependent synaptic machinery. By shifting the balance between kinase-driven processes that support LTP and phosphatase-driven processes that favor LTD, EDC-induced calcium dyshomeostasis alters AMPA receptor trafficking, promotes synaptic weakening, and ultimately impairs memory formation and other higher-order neural functions.

## 7 Mechanistic axis III: oxidative stress and mitochondrial dysfunction

A major convergent downstream mechanism across multiple classes of EDCs is the induction of oxidative stress and mitochondrial dysfunction. Because neuronal survival and synaptic plasticity depend on tightly regulated redox balance, mitochondrial energy production, and calcium buffering, disruption of these processes has profound consequences for synaptic maintenance and neuronal communication.

One important mechanism amplifying EDC-induced neurotoxicity is the excessive generation of reactive oxygen species (ROS). BPA and related compounds increase ROS production, thereby promoting neuronal damage and apoptosis (Kobayashi et al., 2020; Shen et al., 2023). In parallel, these compounds enhance microglial activation, leading to the release of pro-inflammatory cytokines that further destabilize neuronal networks (Kagawa and Nagao, 2020; Zhu et al., 2015). In this way, oxidative and inflammatory processes act synergistically with endocrine signaling disturbances and contribute to persistent functional impairment.

Mycotoxins can also affect synaptic integrity through oxidative stress-related mechanisms. At the neuronal level, ZEN significantly suppresses gene expression in nerve cells, an effect closely linked to the induction of oxidative stress, which is considered a central upstream signal in its neurotoxic action. The overproduction of free radicals damages cellular membranes and mitochondria, thereby triggering apoptosis and impairing neuronal differentiation. Importantly, these effects are not limited to acute toxicity, but may also induce long-term alterations in synaptic plasticity. Exposure to mycotoxins such as ZEN is therefore thought to reduce the brain’s capacity for adaptive synaptic remodeling, ultimately contributing to cognitive dysfunction (Venkataramana et al., 2014).

Pesticides similarly interfere with mitochondrial and redox homeostasis. They disrupt mitochondrial respiratory chain function and energy metabolism, thereby impairing the ATP production required for synaptic maintenance and neurotransmission (Cowie et al., 2017). The organochlorine pesticide dieldrin, for example, damages mitochondrial respiratory chain proteins and increases oxidative stress in the central nervous system. Arsenic likewise exerts neurotoxic effects through ROS generation, disrupted mitochondrial function and Ca^2+^-dependent neuronal cell death (Zsarnovszky et al., 2024). These disturbances are closely linked to synaptic plasticity, as mitochondrial dysfunction compromises energy supply, calcium homeostasis, and the activity of plasticity-related transcription factors such as cAMP response element-binding protein (CREB).

Glial cells are also critically involved in this pathogenic cascade. Astrocytes play an essential role in actin cytoskeleton organization and are therefore indispensable for neuronal migration and synapse maturation (Parent et al., 2011). EDC-induced disruption of glial function can thus have major downstream consequences for synaptic development and stability. Organotin compounds provide a clear example of this mechanism. Dibutyltin activates microglial cells, enhances oxidative stress, and increases the expression of pro-inflammatory cytokines, thereby feeding inflammatory signals into neuronal circuits and contributing to synaptic dysregulation (Chantong et al., 2014). In the neocortex and hippocampus, such inflammatory and oxidative processes are associated with increased apoptosis and a reduction in functional synapses. Tributyltin further exacerbates neuronal vulnerability through additional mechanisms, including inhibition of mitochondrial ATP synthase, disruption of astrocytic glutamate homeostasis, and altered firing frequency of hypothalamic neurons. In parallel, tributyltin chloride has also been linked to blood–brain barrier (BBB) disruption, oxidative stress, and neurodegenerative changes in rat brain (Mitra et al., 2013). Together, these findings show that organotin compounds destabilize glial homeostasis at multiple levels, thereby amplifying neuronal injury and impairing synaptic development and maintenance.

Phthalates also contribute to synaptic dysfunction through oxidative stress and associated mitochondrial impairment. After oral administration, DINP causes brain damage in mice characterized by increased oxidative stress, neuroinflammation, and apoptosis (Peng, 2015). Similarly, DBP induces oxidative stress at higher doses and is associated with anxiety-like behavioral alterations (Shincy and Chitra, 2021). In addition, DBP and its metabolite MBP have been linked to spatial cognitive deficits together with altered hippocampal apoptosis-related proteins such as Bax and caspase-3, although this evidence comes from a co-exposure model with arsenic, so the original MnBP-only wording is too strong (Mao et al., 2016). Importantly, these stress-related effects extend into intracellular signaling pathways directly relevant to synaptic plasticity. BBP has been shown to reduce the activity of the transcription factor CREB, a central regulator of LTP, LTD, and memory formation. Chronic exposure also alters the expression of plasticity-associated proteins, including ERK1/2, MeCP2, and ERα (Betz et al., 2013; Min et al., 2014). Together, these findings indicate that phthalate-induced oxidative stress converges on critical intracellular signaling networks, thereby linking cellular damage to impaired synaptic plasticity and behavioral dysfunction.

Heavy metals represent another major source of oxidative and mitochondrial injury in the nervous system. Arsenic and cadmium induce oxidative damage, mitochondrial dysfunction, and apoptosis, thereby compromising neuronal viability and synaptic integrity (Zsarnovszky et al., 2024). More broadly, these effects reinforce the view that disruption of mitochondrial function and redox regulation is a shared neurotoxic mechanism among chemically diverse EDCs.

Flame-retardant neurotoxicity also extends into oxidative and metabolic pathways that support synaptic plasticity. Prenatal exposure to a mixture of OPFRs altered placental gene programs linked to oxidative-stress defense, including downregulation of Sod2, while developmental organophosphate ester exposure in brain produced transcriptomic signatures enriched for mitochondrial transcriptional regulation. In a complementary prairie vole model, gestational FireMaster 550 exposure disrupted placental monoamine production, particularly serotonin, and altered fetal-brain genes required for cellular respiration. These observations indicate that FRs can compromise both redox buffering and mitochondrial/metabolic support during synaptic development (Marinello et al., 2023; Newell et al., 2023a; Rock et al., 2020).

Similar downstream consequences have also been described for POPs. PAHs and PCBs activate mitochondria-mediated apoptotic pathways, thereby further compromising neuronal survival (Zhang et al., 2016). Across these different EDC classes, oxidative stress and mitochondrial dysfunction converge on key regulators of synaptic plasticity, particularly CREB signaling and BDNF expression. Because both CREB and BDNF are essential for synaptic remodeling, memory consolidation, and long-term neuronal resilience, their impairment represents a critical mechanistic intersection linking cellular stress to enduring deficits in synaptic plasticity and cognitive function.

## 8 Mechanistic axis IV: epigenetic reprogramming

EDCs can induce persistent changes in gene expression through epigenetic mechanisms, thereby providing a plausible basis for the long-term and in some cases intergenerational or transgenerational effects of developmental exposure. Rather than causing only transient endocrine disturbances, these compounds can reprogram DNA methylation, histone modification, and non-coding RNA profiles in ways that durably alter neuronal differentiation, synaptic signaling, and circuit maturation.

Bisphenols are among the best-characterized examples of this mechanism. Exposure during embryonic development alters genomic methylation patterns and thereby modifies long-term gene expression programs. In mice, BPA exposure has been shown to alter the epigenomic profile of the fetal forebrain (Yaoi et al., 2008). Comparable associations have also been reported in humans, where prenatal BPA exposure was associated with altered DNA methylation of the glutamate receptor subunit gene GRIN2B in 7-year-old girls (Alavian-Ghavanini et al., 2018). BPA further increases expression of DNA methyltransferase 1 in the amygdala, a change associated with increased anxiety-like behavior in rat offspring (Zhou et al., 2013). In addition, the delayed developmental shift of GABAergic signaling from excitatory to inhibitory in cortical neurons has been linked to epigenetic modifications induced by BPA (Yeo et al., 2013).

Phthalates likewise exert important epigenetic effects. They alter histone deacetylase expression and microRNA profiles, indicating disruption of neurodevelopmental programming at multiple regulatory levels. Postnatal exposure to DEHP has been shown to induce sex-specific changes in hippocampal microRNA expression. In particular, downregulation of miR-93 together with upregulation of TNFAIP1 promotes degradation of protein kinase CK2β, leading to inhibition of the Akt/CREB signaling pathway and ultimately neuronal cell death. These epigenetically mediated alterations converge directly on key intracellular pathways involved in synaptic plasticity and neuronal survival (López-Merino et al., 2022; Qiu et al., 2023). Additional evidence suggests broader dysregulation of the epigenetic machinery by phthalates, including altered expression of histone deacetylases such as HDAC4 and HDAC5, supporting the view that chromatin remodeling is a central component of their neurotoxic action (Guida et al., 2014).

POPs such as PCBs also exhibit epigenetic activity. PCBs have been associated with global DNA hypomethylation and altered microRNA expression, particularly in the developing brain. Age- and sex-specific changes in microRNA expression have also been described in the developing hypothalamus of rats, suggesting that PCB exposure may reprogram neuroendocrine and synaptic development through long-lasting gene-regulatory mechanisms (Dickerson et al., 2011; Topper et al., 2015). These effects complement the receptor-mediated, calcium-dependent, and oxidative mechanisms described above and may help explain the unusually persistent neurobehavioral consequences of PCB exposure.

Pesticides should also be considered within this epigenetic framework. Chronic exposure during sensitive developmental windows can induce stable changes in DNA methylation, histone modification, and miRNA expression. Such alterations affect genes that are crucial for synaptic transmission and plasticity, thereby extending the impact of exposure far beyond the initial toxic insult. This epigenetic reprogramming provides a biologically plausible explanation for the observation that pesticide-induced neurotoxic effects may persist long after exposure and, in some cases, may also be transmitted to subsequent generations.

Direct evidence that FRs induce classical epigenetic remodeling remains less developed than for bisphenols or phthalates, but recent developmental studies indicate durable gene-regulatory reprogramming. Organophosphate ester exposure produced sex-specific hippocampal transcriptomic signatures involving mitochondrial regulation and cation transport, while gestational FireMaster 550 exposure altered placental and fetal-brain developmental gene programs in a dose- and sex-dependent manner. These data support the interpretation that FRs can leave persistent transcriptional imprints on neurodevelopmental pathways, even when DNA methylation or histone marks were not directly measured (Marinello et al., 2023; Newell et al., 2023a).

Taken together, these findings support epigenetic reprogramming as a major mechanistic axis of EDC-induced synaptic dysfunction. By altering the regulatory architecture that controls neuronal development, plasticity-related signaling, and synaptic gene expression, EDCs create a substrate for enduring functional impairment. Importantly, because epigenetic marks can outlast the original exposure and in some contexts be propagated across generations, this axis provides the strongest mechanistic bridge between early-life exposure and persistent or inherited neurobehavioral abnormalities.

## 9 Mechanistic axis V: network-level and barrier dysfunction

Beyond their molecular and synaptic actions, EDCs can also destabilize brain homeostasis at the level of the neurovascular unit and neural networks. This dimension is particularly important because BBB integrity and glia–neuron interactions determine whether local cellular insults remain spatially restricted or propagate into broader circuit dysfunction. Phthalates provide a clear example of this process. In adult male mice, six-week oral exposure to low doses of DEHP alone or in an environmental phthalate mixture increased BBB permeability in the hypothalamic medial preoptic area and in the hippocampal CA1 and CA3 regions, accompanied by altered expression of the tight-junction-associated protein zona occludens-1 and caveolae protein Cav-1, as well as local glial activation (Ahmadpour et al., 2021; Damiano et al., 2025). Such barrier impairment is mechanistically significant because it facilitates the entry of peripheral immune mediators and other harmful substances into vulnerable brain regions, thereby reinforcing neuroinflammatory and neurotoxic cascades (Ahmadpour et al., 2021; Damiano et al., 2025).

Network-level dysfunction is further amplified by persistent activation of microglia and astrocytes. Microglia are essential for synaptic pruning and circuit refinement during development, but chronic inflammatory activation can shift them toward maladaptive synapse elimination and aberrant remodeling. This provides a plausible link between EDC-induced neuroinflammation and large-scale disturbances in connectivity and plasticity (Lara de Deus et al., 2025; Yang et al., 2025). Consistent with this framework, multiple EDC classes including phthalates, BPA, PCBs, chlorpyrifos, and organotins have been associated in animal studies with hypothalamic or hippocampal inflammation, microglial activation, and altered inflammatory signaling. Organotin compounds such as tributyltin appear particularly disruptive in this respect, as they have been linked to BBB breakdown, astrocyte activation, hypothalamic inflammation, and neurodegeneration in several brain regions (Stathori et al., 2024).

Recent flame-retardant studies also support network-level disruption extending beyond individual synapses. Gestational FireMaster 550 exposure disrupted the placenta–brain axis, with dose- and sex-specific changes in placental monoamine production and fetal-brain axon-guidance programs, while perinatal FM 550 exposure altered social-brain targets in a sex- and chemical-specific manner, including oxytocin receptor- and serotonin-related elements in regions such as the amygdala and BNST. In addition, developmental FM 550 exposure changed intrinsic membrane properties of nucleus accumbens core medium spiny neurons, showing reduced input resistance in both sexes. Together, these findings indicate that FRs destabilize circuit-level excitability and the developmental organization of socially relevant neural networks (Krentzel et al., 2021; Marinello et al., 2023; Schkoda et al., 2024).

Astrocytes are equally central to this pathogenic axis. Under physiological conditions, they maintain BBB function, regulate extracellular ion balance, recycle neurotransmitters such as glutamate, provide metabolic support to neurons, and participate in synapse formation and elimination. When driven into a reactive state by EDC-associated inflammation and oxidative stress, however, astrocytes can lose these homeostatic functions and instead sustain chronic inflammatory signaling, impair neurotransmitter clearance, and weaken neuronal metabolic support (Calì et al., 2024; Khakh, 2025; Rupareliya et al., 2023). Taken together, disruption of barrier integrity, glial homeostasis, and circuit refinement provides a mechanistic bridge between local synaptic lesions and systems-level dysfunction, helping to explain how chemically diverse EDCs ultimately produce widespread deficits in cognition, emotional regulation, and neuroendocrine control.

## 10 Behavioral and cognitive outcomes

Behavioral and cognitive outcomes are among the most consequential manifestations of EDC-induced synaptic dysfunction. At the functional level, molecular and synaptic disturbances translate into impaired learning and memory, anxiety- and mood-related alterations, and neurodevelopmental abnormalities, including ADHD- and autism-like behaviors. Across human cohorts and experimental models, phthalates, PCBs, dioxins, pesticides, and FRs have been associated with persistent impairments in cognition, attention, emotional regulation, motor performance, and sex-specific neurobehavioral development. These phenotypes are consistent with the mechanistic disturbances described above and, in selected cases, extend beyond the directly exposed generation.

Some of the clearest links between synaptic disruption and behavioral dysfunction have been demonstrated for pesticides and FRs. Perinatal exposure to low doses of chlordane or chlorpyrifos oxon (CPO) caused abnormalities in motor and cognitive development in rats. CPO increased pupillary weight and prolonged the latency of the pupillary reflex, indicating delayed motor maturation. Notably, the attenuation of mGluR-LTD induced by CPO and chlordane persisted into adulthood despite preserved basal synaptic transmission. This selective impairment of synaptic plasticity translated into deficits in object familiarity memory and spatial orientation, with chlordane impairing spatial memory in a dose-dependent manner (López-Merino et al., 2022). Other pesticides likewise produce marked behavioral changes. Exposure to dicofol has been associated with long-lasting cognitive and emotional impairments, while prenatal exposure to mirex has been linked to neurodevelopmental disorders in children evident as early as preschool age (Jin and Karlstedt, 2025; Puertas et al., 2010). These mechanistic abnormalities are likewise mirrored by behavioral findings for FRs. Phosphate-based FRs, which downregulate synaptic structural proteins such as PSD-95, impair spatial learning in the Morris Water Maze, a classic test of hippocampus-dependent plasticity (Rojas et al., 2025). More specifically, developmental exposure to TPP caused long-lasting neurobehavioral and neurochemical dysfunction, consistent with persistent disruption of dopaminergic and broader monoaminergic signaling (Hawkey et al., 2023). In prairie voles, perinatal FM 550 exposure altered partner preference in a sex-specific manner, enhancing partner preference in females but decreasing it in males, and this phenotype was accompanied by reduced input resistance in nucleus accumbens core medium spiny neurons (Krentzel et al., 2021). Recent work in Wistar rats further indicates sex- and chemical-specific disruption of oxytocin receptor- and serotonin-related elements of the social-brain network after perinatal FM 550 or component exposure, providing a plausible circuit-level substrate for altered socioemotional behavior (Schkoda et al., 2024). Together, these findings show that flame retardant-induced disturbances at the synaptic, electrophysiological, and neuromodulatory levels are translated into persistent deficits in learning, motivation, and social behavior.

Human epidemiological studies also provide substantial evidence for adverse neurobehavioral outcomes after phthalate exposure. Prenatal exposure to phthalate metabolites has been associated with impaired cognitive development. Girls exposed to higher concentrations of high-molecular-weight phthalate metabolites showed significantly lower orientation scores on the Brazelton Neonatal Behavioral Assessment Scale, indicating deficits in attention, visual and auditory stimulus processing, and alertness within the first days of life (Engel et al., 2009). Sex-dependent effects have also been reported, particularly in motor development, which differed according to the specific phthalate involved (Schug et al., 2015; Weiss, 2011). Increased phthalate exposure has further been associated with neurocognitive problems in later childhood (Bettio et al., 2020; Dias et al., 2025). A supralinear association between MnBP exposure and deficits in social behavior, cognition, and attention has also been described (Won et al., 2016). Prenatal phthalate exposure has been linked to ADHD-like symptoms, and studies from Sweden suggest that DINP contributes to sex-specific differences in play behavior, being associated with reduced masculine play behavior in boys (Özel et al., 2023; Swan et al., 2010). Another cohort study reported an association between phthalate exposure and autism-like behaviors in children aged 7–9 years (Engel et al., 2010).

Animal studies complement these epidemiological observations and provide mechanistic support for phthalate-induced behavioral dysfunction. In rodents, DBP impairs memory performance, reduces motor activity, and induces anxiety-like behavior (Farzanehfar et al., 2016), while DINP similarly causes anxiety and cognitive deficits (Ma et al., 2015). Perinatal exposure to DBP or DEHP also alters sexual behavior and modifies hypothalamic gene expression in rats (Hunter et al., 2021; Lee et al., 2006). These findings strengthen the interpretation that phthalate-induced disturbances in synaptic signaling and neuroendocrine development are translated into stable behavioral abnormalities.

Similarly robust evidence exists for PCB- and dioxin-related neurobehavioral impairment. Numerous cohort studies from Taiwan, Michigan, New York, the Netherlands, Germany, and the Faroe Islands have documented adverse effects of prenatal PCB exposure on cognitive function, learning ability, memory performance, and intelligence quotient in children (Jacobson and Jacobson, 2003, 1996; Lai et al., 2001). Reduced attention span and increased impulsivity—features reminiscent of ADHD—were reported particularly in Dutch and American cohorts (Jacobson and Jacobson, 2003). Sex-specific effects have also been described, for example in Taiwan, where prenatally exposed boys showed deficits in spatial abilities (Schug et al., 2015). In addition, prenatal exposure to CB-118 was negatively correlated with motor development and expressive language skills in children. Experimental studies parallel these epidemiological findings: gestational exposure to PCB- and dioxin-like compounds reduces learning and memory performance and impairs hippocampus-dependent synaptic plasticity in rodents. In workers occupationally exposed to TCDD, neurological and neurophysiological abnormalities were detected even decades after exposure, suggesting that the functional consequences of these pollutants may be exceptionally long-lasting (Pelclova et al., 2018; Pelclová et al., 2001).

Particularly important are the observations that some neurobehavioral effects persist across generations. In the case of phthalates, DEHP exposure during pregnancy and lactation alters the expression of pituitary hormones and hypothalamic–pituitary–adrenal axis-related stress hormones across generations, and this is accompanied by social behavioral abnormalities in descendants that were not directly exposed (Hatcher et al., 2019; Quinnies et al., 2017). A related study showed that female offspring of the third generation (F3) exhibited reduced anxiety after ancestral high-dose DEHP exposure, whereas no such effect was observed in males; this sex-specific difference correlated with reduced ERα expression in the amygdala of F3 females (Dias et al., 2025). These findings provide some of the strongest behavioral evidence that developmental EDC exposure can produce true transgenerational neurobehavioral effects. PCB and dioxin exposure likewise induces enduring consequences anchored in endocrine and epigenetic dysregulation. Prenatal PCB exposure disrupts early hypothalamic neuroendocrine development in rats, resulting in reduced thyroid hormone levels and consequent motor deficits and sensory impairments such as hearing loss; notably, these abnormalities can be mitigated by thyroxine replacement therapy, supporting a causal role for thyroid disruption (Dickerson et al., 2011; Goldey and Crofton, 1998). Dioxins and furans also exert antiandrogenic effects (Weiss, 2011, 2007), potentially amplifying hormonal imbalance during critical windows of brain development. Although the evidence for true transgenerational inheritance is stronger for some chemical classes than for others, the available data collectively indicate that EDC-induced behavioral dysfunction can be strikingly persistent and, under certain conditions, extend across generations.

Collectively, these findings show that chemically diverse EDCs produce convergent behavioral phenotypes despite marked heterogeneity in structure and primary molecular targets. Human cohort studies and animal models consistently point to impairments in learning, memory, attention, emotional regulation, motor behavior, and sex-specific neurodevelopment. The persistence of these outcomes across the lifespan and in selected models across generations strongly supports the view that disruptions in receptor signaling, calcium homeostasis, oxidative stress pathways, mitochondrial function, and epigenetic regulation ultimately translate into stable behavioral and cognitive dysfunction.

## 11 Conclusion and future perspectives

Taken together, the evidence reviewed here shows that chemically diverse EDCs converge on a limited set of core mechanisms that regulate synaptic plasticity. Despite marked differences in structure and primary molecular targets, these compounds repeatedly disrupt receptor-mediated signaling, excitatory and inhibitory neurotransmission, calcium-dependent kinase and phosphatase pathways, mitochondrial function, oxidative homeostasis, epigenetic programming, and glia- and barrier-dependent network stability. In this way, heterogeneous EDCs converge on shared nodes of synaptic regulation including NMDA receptor/AMPA receptor signaling, Ca^2+^ dynamics, ERK/CREB-dependent transcription, and structural synaptic maintenance thereby shifting the balance between LTP and LTD and impairing learning, memory, and behavior.

A central conclusion of this review is therefore that a mechanistic framework provides a more coherent and predictive understanding of EDC-induced neurotoxicity than traditional substance-based classifications. Rather than viewing bisphenols, phthalates, pesticides, PCBs, dioxins, heavy metals, FRs, and related compounds as isolated toxicological categories, the present synthesis shows that they act through overlapping biological pathways that ultimately destabilize synaptic integrity and cognitive function. This convergence is particularly important because it explains why chemically distinct exposures can produce similar neurobehavioral phenotypes, including deficits in memory, attention, emotional regulation, motor function, and neurodevelopment.

The reviewed evidence also indicates that EDC-induced disruption of synaptic plasticity is not limited to acute or transient effects. By coupling receptor and calcium disturbances to oxidative stress, mitochondrial injury, glial activation, epigenetic reprogramming, and, in some cases, BBB dysfunction, these compounds can generate persistent maladaptive changes in neural circuits. This multi-level persistence helps explain the long-term nature of many observed cognitive and behavioral effects and, in selected models, their extension across generations.

From a broader perspective, the mechanism-centered view developed here may also have practical value. It highlights key nodes of vulnerability such as glutamatergic receptor signaling, intracellular calcium homeostasis, mitochondrial resilience, CREB/BDNF-associated transcriptional control, and epigenetic regulation that may serve as useful targets for future toxicological testing, biomarker development, and therapeutic intervention. In parallel, it supports the argument that regulatory assessment should move beyond isolated compound-specific endpoints and incorporate convergent neurobiological mechanisms that are shared across EDC classes.

Future research should therefore focus on integrating multi-level evidence, linking molecular signaling, synaptic structure and physiology, network function, and behavioral outcomes within unified experimental and translational models. Greater emphasis should also be placed on sensitive developmental windows, low-dose and mixture effects, sex-specific vulnerability, and the distinction between persistent, intergenerational, and truly transgenerational consequences. Finally, the identification of robust biomarkers of EDC-induced synaptic dysfunction whether molecular, electrophysiological, imaging-based, or behavioral will be essential for improving early detection and risk stratification. A deeper understanding of these convergent mechanisms is crucial for mitigating the long-term neurological risks posed by environmental chemical exposure.
